# Feasibility and Acceptability of Pediatric Smartphone Lung Auscultation by Parents: Cross-Sectional Study

**DOI:** 10.2196/52540

**Published:** 2024-04-08

**Authors:** Catarina Santos-Silva, Henrique Ferreira-Cardoso, Sónia Silva, Pedro Vieira-Marques, José Carlos Valente, Rute Almeida, João A Fonseca, Cristina Santos, Inês Azevedo, Cristina Jácome

**Affiliations:** 1 Faculty of Medicine, University of Porto, Porto, Portugal; 2 Department of Pediatrics, Centro Hospitalar Universitário de São João, Porto, Portugal; 3 CINTESIS - Center for Health Technology and Services Research, Faculty of Medicine, Universidade do Porto, Porto, Portugal; 4 MEDIDA – Serviços em Medicina, Educação, Investigação, Desenvolvimento e Avaliação, Porto, Portugal; 5 CINTESIS@RISE, Department of Community Medicine, Information and Health Decision Sciences (MEDCIDS), Faculty of Medicine, University of Porto, Porto, Portugal; 6 Department of Obstetrics, Gynecology and Pediatrics, Faculty of Medicine, Universidade do Porto, Porto, Portugal; 7 EpiUnit, Institute of Public Health, Universidade do Porto, Porto, Portugal

**Keywords:** respiratory sounds, respiratory, respiration, lung, lungs, pulmonary, breathing, sound, sounds, wheeze, crackle, child, children, pediatric, pediatrics, parent, parents, parenting, asthma, auscultation, smartphone, mobile applications, mHealth, mobile health, app, apps, applications, crackles, wheezes, wheezing, participation, patient participation, willingness, adoption, acceptance, usability, attitude, attitudes, opinion, perception, perceptions, smartphone, smartphones, intent, ease of use, survey, surveys, questionnaire, questionnaires, mobile phone

## Abstract

**Background:**

The use of a smartphone built-in microphone for auscultation is a feasible alternative to the use of a stethoscope, when applied by physicians.

**Objective:**

This cross-sectional study aims to assess the feasibility of this technology when used by parents—the real intended end users.

**Methods:**

Physicians recruited 46 children (male: n=33, 72%; age: mean 11.3, SD 3.1 y; children with asthma: n=24, 52%) during medical visits in a pediatric department of a tertiary hospital. Smartphone auscultation using an app was performed at 4 locations (trachea, right anterior chest, and right and left lung bases), first by a physician (recordings: n=297) and later by a parent (recordings: n=344). All recordings (N=641) were classified by 3 annotators for quality and the presence of adventitious sounds. Parents completed a questionnaire to provide feedback on the app, using a Likert scale ranging from 1 (“totally disagree”) to 5 (“totally agree”).

**Results:**

Most recordings had quality (physicians’ recordings: 253/297, 85.2%; parents’ recordings: 266/346, 76.9%). The proportions of physicians' recordings (34/253, 13.4%) and parents' recordings (31/266, 11.7%) with adventitious sounds were similar. Parents found the app easy to use (questionnaire: median 5, IQR 5-5) and were willing to use it (questionnaire: median 5, IQR 5-5).

**Conclusions:**

Our results show that smartphone auscultation is feasible when performed by parents in the clinical context, but further investigation is needed to test its feasibility in real life.

## Introduction

Respiratory diseases are leading causes of morbidity and mortality worldwide, and infants and young children are particularly vulnerable. Asthma is the most common chronic respiratory disease in children, affecting approximately 14% of children globally, and its prevalence is increasing [1,2]. Health care costs associated with respiratory diseases are increasing burdens on the global economy, including primary and inpatient health care costs; disability-adjusted life-years; and lost productivity, with a high number of school and work days lost [3,4].

Remote monitoring strategies can play an important role in controlling symptoms, improving patients’ quality of life, and detecting adverse events that are associated with significant morbidity [5,6]. Lung auscultation is a quick, inexpensive, and efficient way to assess the respiratory system and help monitor a child’s respiratory status [7,8]. However, as auscultation with a stethoscope is commonly performed by a physician during in-person visits, there is a need for suitable alternatives that support teleconsultation and empower families to take control of their own health and others’ health.

As the ownership of a smartphone is now extremely common, Reyes et al [8] and Ferreira-Cardoso et al [9] recently proposed using a smartphone to acquire lung sounds; the former used an electret microphone connected to a smartphone, and the latter used smartphone built-in microphones. Ferreira-Cardoso et al [9] showed that smartphone auscultation performed by a physician was feasible in children. However, it remains unknown whether smartphone auscultation would be feasible if performed by parents and whether they would accept the use of this technology outside the clinical setting [9,10].

The primary aim of our study was to compare the feasibility of smartphone auscultation when performed by parents versus physicians. As a secondary aim, we evaluated the acceptability and ease of use of this technology among parents.

## Methods

### Ethical Considerations

This study was approved by the ethics committee of Centro Hospitalar Universitário de São João (approval number: 316/20; September 18, 2020). This study was reported in accordance with the recommendations of the STROBE (Strengthening the Reporting of Observational Studies in Epidemiology) Initiative [11]. Written informed consent was obtained from the parents, and assent was obtained from the children. In accordance with national legislation, written informed consent was also obtained from the children if they were aged 16 years or older.

### Study Design

We conducted a cross-sectional study with a convenience sample of children who were followed at the pediatrics department of the Centro Hospitalar Universitário de São João, a tertiary care public hospital in Porto, Portugal. This study took place between December 2022 and May 2023. During medical visits, the physicians invited the children and their parents to participate in this study.

### Participants

Children aged 5 to 17 years, with or without a respiratory disease (eg, asthma, cystic fibrosis, and other respiratory diseases), were included if they had a scheduled medical visit. The exclusion criteria were refusal to participate in this study and any health status or condition that interfered with the correct and safe collection of the children’s lung sounds.

### Data Collection

Data on children’s sex, age, height, weight, and diagnosis were registered in a paper case report form. BMIs were calculated from the anthropometric data.

**Figure 1.**
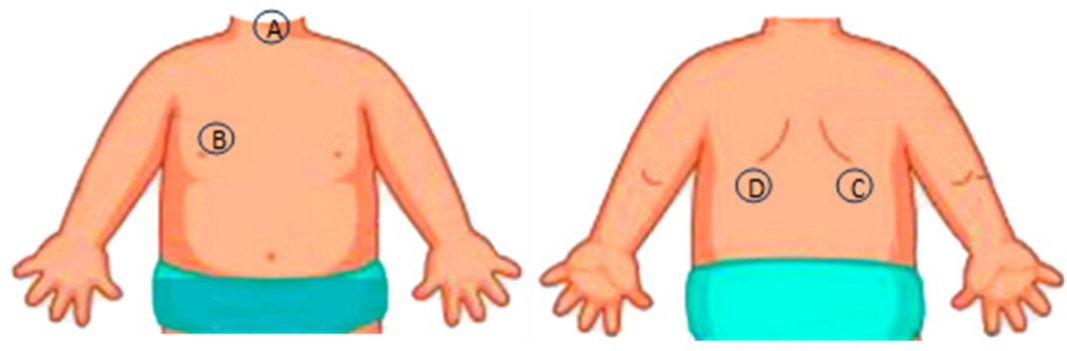
Lung auscultation locations: (A) trachea, (B) right anterior chest, (C) right posterior base, and (D) left posterior base.

Smartphone lung auscultation was performed by the physicians, using the AIRDOC mobile app [10]. The app has multiple features, but only the lung auscultation feature was used for this study. A full description of the app can be found elsewhere [9,10]. During auscultation, the child was seated in an upright position. Auscultation was performed at the following four locations: the three minimum locations recommended for computerized respiratory sound analysis [12]—the trachea and the right and left posterior lung bases—and the right anterior chest, which is known to give a better sense of the presence of adventitious sounds in children (Figure 1) [13,14].

Two 10-second recordings were made at each location, allowing for the inclusion of 1 to 5 respiratory cycles per recording [15]; 2 physicians (ie, 1 physician and 1 final-year medical student) were responsible for making the recordings, using their respective smartphones—an Apple iPhone XR (Apple Inc) and an Apple iPhone 14 (Apple Inc).

After the physicians completed the smartphone auscultations, the parents were invited to participate and were briefly instructed to perform the auscultation by using the same procedures and the same smartphones used by the physicians. Afterward, the parents completed a 3-item questionnaire to provide feedback on the app (app’s ease of use for recording lung sounds, willingness to use the app to send lung sounds to the physician, and recommendation to others), using a Likert scale ranging from 1 (“totally disagree”) to 5 (“totally agree”).

### Lung Sound Recording Classification

Each lung sound recording was listened to independently by 3 annotators—a physiotherapist and lung sound expert (CJ), a medical doctor (HFC), and a final-year medical student (CSS)—using Adobe Audition 2023 version 23.2 (Adobe Inc) and high-quality headphones (Marshall Major IV [‎Zound Industries], SONY Wh-H910Nl [Sony Group Corporation], and Sennheiser HD 380 Pro [Sennheiser electronic GmbH & Co. KG]). They also performed an analysis of sound spectrograms according to the default parameters of Adobe Audition. The three annotators were blinded to all data collected except child IDs and auscultation locations. Despite the shorter duration (10 s instead of the recommended 15 s), the quality of each lung sound recording was assessed according to the European Respiratory Society’s criteria for sounds with quality (ie, minimal artifacts, visible respiratory phases, and a sound of interest could be demonstrated) [16]. The final decision on the quality of recordings was made by consensus among the three annotators. The next step was to evaluate only the lung sound recordings with quality in terms of the presence of adventitious sounds, namely, crackles and wheezes [17]. The final decision as to whether adventitious sounds were present was made by majority rule.

### Data Analysis

Descriptive statistics were used to characterize the participants (ie, sex, age, weight, height, BMI, and diagnosis group [asthma, other respiratory disease, and no respiratory disease]). Shapiro-Wilk tests were used to assess the normality of the data. To explore the existence of differences among the three diagnostic groups, a chi-square test (sex), Kruskal-Wallis test (weight and BMI), and 1-way ANOVA (age and height) were applied.

The proportions of agreement and the proportions of specific agreement (specific agreement for each category) among the three annotators were calculated. Afterward, their interrater reliability was determined by using the Fleiss κ and its 95% CI. The Fleiss κ was interpreted as follows: 0 to 0.20 indicated slight agreement, 0.21 to 0.40 indicated fair agreement, 0.41 to 0.60 indicated moderate agreement, 0.61 to 0.80 indicated substantial agreement, and 0.81 to 1.0 indicated almost perfect agreement [18]. This was done for each auscultation group (ie, physicians and parents) and for each location. Differences in agreement among locations were explored with chi-square tests, and Bonferroni correction was used where necessary.

The proportions of quality recordings and recordings with adventitious sounds were calculated for each auscultation location and auscultation group. Chi-square tests, in which Bonferroni correction was used when necessary, were applied to assess differences among locations. All main analyses were based on the recordings, which were the de facto subjects of analysis in this paper. However, a secondary, more clinically oriented analysis was carried out in parallel by calculating the proportions of participants with at least 1 lung sound recording with quality and at least 1 lung sound recording with adventitious sounds. This was also done for each location and each auscultation group.

The statistics software used was IBM SPSS Statistics (version 28.0.0.0; IBM Corp). R software was used to compute the proportions of agreement with the “obs. Agree” package (R Foundation for Statistical Computing). The level of significance was set at .05.

## Results

### Participants’ Characteristics

**Figure 2.**
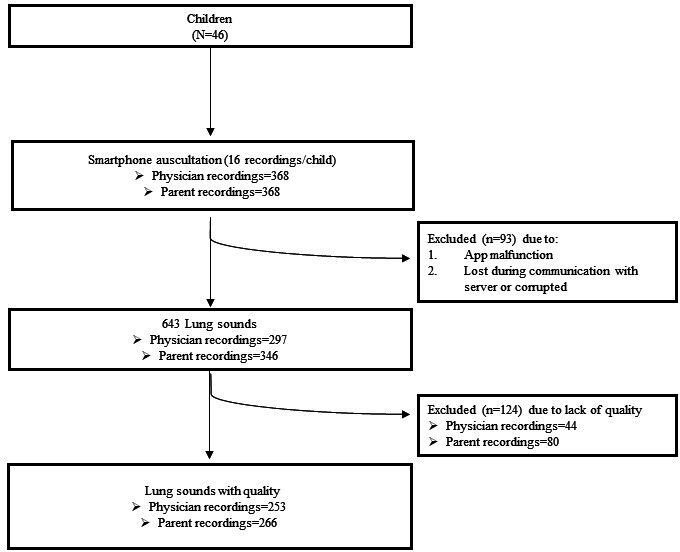
Flowchart showing the number of lung sound recordings and children considered throughout this study.

A total of 46 children were recruited. Most were male (n=33, 72%) and had asthma (n=24, 52%). The mean age was 11.3 (SD 3.1) years. The median BMI was 19.4 (IQR 16.4-21.5) kg/m^2^. The characteristics of the participants are presented in Multimedia Appendix 1. A total of 736 recordings were expected from the 46 children (16 recordings/child), but due to some data losses, 641 (87.1%) recordings from 45 children were analyzed (Figure 2). All 45 children had recordings from both physicians and parents for each location except the right anterior chest, which was missed by the physician for one child and by the parent for another.

### Lung Sound Quality: Agreement and Proportion

The proportion of agreement among the three annotators regarding the quality of the physicians’ lung sound recordings (91%) was similar to that for the quality of parents’ lung sound recordings (85%), corresponding to a Fleiss κ of 0.66 (95% CI 0.59-0.72) and 0.57 (95% CI 0.51-0.63), respectively (Table 1). With regard to the physicians’ recordings, agreement for recordings taken at the trachea was greater than that for recordings taken at the right anterior and posterior locations (Multimedia Appendix 2). No statistically significant differences in the agreement among locations were found in the parents’ recordings (Multimedia Appendix 2).

**Table 1.** Interrater reliability and proportions of agreement (PAs) among experts on the lung sound quality of physicians’ and parents’ lung sound recordings.

Location and quality of recordings		Physicians’ recordings		Parents’ recordings	
		Fleiss κ (95% CI)	PA (95% CI)	Fleiss κ (95% CI)	PA (95% CI)
**Trachea**		0.50 (0.37-0.63)	0.99 (0.97-1.00)	0.51 (0.39-0.63)	0.90 (0.84-0.95)
	No quality	N/A^a^	0.50 (0.50-0.50)	N/A	0.57 (0.37-0.73)
	Quality	N/A	0.99 (0.99-1.00)	N/A	0.94 (0.91-0.97)
**Right anterior chest**		0.58 (0.45-0.71)	0.84 (0.77-0.90)	0.51 (0.38-0.63)	0.81 (0.74-0.86)
	No quality	N/A	0.68 (0.53-0.80)	N/A	0.63 (0.51-0.74)
	Quality	N/A	0.90 (0.84-0.94)	N/A	0.87 (0.82-0.91)
**Right posterior base**		0.54 (0.40-0.67)	0.88 (0.82-0.94)	0.60 (0.47-0.72)	0.82 (0.75-0.88)
	No quality	N/A	0.61 (0.37-0.76)	N/A	0.73 (0.61-0.82)
	Quality	N/A	0.93 (0.89-0.96)	N/A	0.87 (0.80-0.92)
**Left posterior base**		0.81 (0.68-0.94)	0.94 (0.90-0.98)	0.58 (0.46-0.70)	0.88 (0.82-0.93)
	No quality	N/A	0.84 (0.70-0.94)	N/A	0.65 (0.48-0.79)
	Quality	N/A	0.97 (0.93-0.99)	N/A	0.93 (0.89-0.96)
**All locations**		0.66 (0.59-0.72)	0.91 (0.89-0.94)	0.57 (0.51-0.63)	0.85 (0.82-0.88)
	No quality	N/A	0.71 (0.62-0.78)	N/A	0.66 (0.59-0.72)
	Quality	N/A	0.95 (0.93-0.97)	N/A	0.90 (0.88-0.92)

a N/A: not applicable.

The proportion of quality recordings was high—85.2% (253/297) for recordings obtained by physicians and 76.9% (266/346) for recordings obtained by parents (Table 2). The proportion of quality recordings at the trachea was statistically superior to all other locations for the physicians’ recordings and superior to the right posterior base for the parents’ recordings (Multimedia Appendix 2). When using the participants (n=45) as the unit of analysis, the majority had at least 1 lung sound recording with quality per location when recordings were acquired by the physicians (n=30, 67%) and when they were acquired by parents (n=27, 60%).

**Table 2.** Proportions of physicians’ lung sound recordings and parents’ lung sound recordings with quality.

Location of recordings	Quality lung sound recordings, n/N (%)	
	Physicians’ recordings	Parents’ recordings
Trachea	75/76 (98.7)	64/74 (86.5)
Right anterior chest	57/76 (75)	53/75 (70.7)
Right posterior base	63/73 (86.3)	48/70 (68.6)
Left posterior base	58/72 (80.6)	59/70 (84.3)
All locations	253/297 (85.2)	266/346 (76.9)

### Adventitious Sounds: Agreement and Proportion

The proportion of agreement among the three annotators regarding the presence of adventitious sounds was 91% (κ=0.60, 95% CI 0.53-0.68) for recordings obtained by physicians and 91% (κ=0.62, 95% CI 0.55-0.70) for those obtained by parents (Table 3). No statistically significant differences in the agreement were seen when considering locations separately (all *P* values were >.05).

**Table 3.** Interrater reliability and proportions of agreement (PAs) among experts on the presence of adventitious sounds in the physicians’ and parents’ lung sound recordings.

Recording location and presence of adventitious sounds		Physicians’ recordings		Parents’ recordings	
		Fleiss κ (95% CI)	PA (95% CI)	Fleiss κ (95% CI)	PA (95% CI)
**Trachea**		0.63 (0.49-0.76)	0.91 (0.86-0.95)	0.45 (0.31-0.59)	0.86 (0.78-0.92)
	Absent	N/A^a^	0.94 (0.91-0.98)	N/A	0.92 (0.87-0.95)
	Present	N/A	0.68 (0.45-0.83)	N/A	0.53 (0.25-0.71)
**Right anterior chest**		0.75 (0.59-0.91)	0.95 (0.89-0.99)	0.63 (0.48-0.79)	0.87 (0.79-0.94)
	Absent	N/A	0.97 (0.94-0.99)	N/A	0.92 (0.86-0.96)
	Present	N/A	0.78 (0.50-0.95)	N/A	0.71 (0.52-0.85)
**Right posterior base**		0.51 (0.35-0.66)	0.88 (0.82-0.95)	0.73 (0.57-0.90)	0.94 (0.88-0.99)
	Absent	N/A	0.93 (0.89-0.97)	N/A	0.97 (0.93-0.99)
	Present	N/A	0.57 (0.33-0.76)	N/A	0.76 (0.44-0.95)
**Left posterior base**		0.52 (0.37-0.67)	0.92 (0.86-0.98)	0.75 (0.61-0.90)	0.97 (0.92-1)
	Absent	N/A	0.95 (0.91-0.99)	N/A	0.98 (0.96-1)
	Present	N/A	0.56 (0.21-0.79)	N/A	0.77 (0.22-1)
**All locations**		0.60 (0.53-0.68)	0.91 (0.88-0.94)	0.62 (0.55-0.70)	0.91 (0.88-0.93)
	Absent	N/A	0.95 (0.93-0.97)	N/A	0.95 (0.93-0.96)
	Present	N/A	0.65 (0.54-0.75)	N/A	0.67 (0.55-0.77)

a N/A: not applicable.

Adventitious sounds were found in 13.4% (34/253) and 11.7% (31/266) of the recordings obtained by physicians and parents, respectively (Table 4). Comparisons between auscultation locations showed no significant statistical differences (all *P* values were >.05).

**Table 4.** Proportions of physicians’ lung sound recordings and parents’ lung sound recordings with adventitious sounds.

Location of recordings	Recordings with adventitious sounds, n/N (%)	
	Physicians’ recordings	Parents’ recordings
Trachea	12/75 (16)	8/64 (12.5)
Right anterior chest	7/57 (12.3)	9/53 (17)
Right posterior base	10/63 (15.9)	4/48 (8.3)
Left posterior base	5/58 (8.6)	3/59 (5.1)
All locations	34/253 (13.4)	31/266 (11.7)

When using the participants (n=45) as the unit of analysis, 19 (42%) participants had at least 1 lung sound recording with adventitious sounds (19 such recordings were obtained by the physician, and 17 such recordings were obtained by the parents).

### Parents’ App Feedback

As can be seen in Table 5, overall, the parents would recommend the app to others (questionnaire: median 5, IQR 5-5), found it easy to work with (questionnaire: median 5, IQR 5-5), and were willing to use it to send lung sounds to the physician (questionnaire: median 5, IQR 5-5).

**Table 5.** Parents’ (n=45; 1 missing response) feedback about the app.

Questionnaire item	App feedback, n (%)				
	Totally agree	Agree	Neither agree nor disagree	Disagree	Totally disagree
App’s ease of use for recording lung sounds	39 (87)	4 (9)	0 (0)	1 (2)	1 (2)
Willing to use the app to send lung sounds to the physician	38 (84)	3 (7)	3 (7)	0 (0)	1 (2)
Would recommend the app to others	38 (84)	5 (11)	1 (2)	0 (0)	1 (2)

## Discussion

### Principal Results

To our knowledge, this is the first study to assess the feasibility and acceptability of parents using a smartphone built-in microphone to capture lung sounds.

We found similar results when comparing the proportion of parents’ quality recordings (266/346, 76.9%) with the proportion of physicians’ quality recordings in this study (253/297, 85.2%) and that in a study by Ferreira-Cardoso et al [9] (73%). The same could be said for recordings with adventitious sounds; the proportion of parents’ recordings with adventitious sounds (31/266, 11.7%) was similar to the proportion of physicians’ recordings with adventitious sounds in this study (34/253, 13.4%) and that in the study by Ferreira-Cardoso et al [9] (14%).

Naturally, we attributed some of the differences in quality and adventitious sound proportion to the fact that the instructions on how to use the app were brief and the fact that parents were inexperienced and therefore misplaced the smartphone, pressed too gently, or even moved the smartphone or talked during the recordings.

Lung sounds were also recorded in a quiet but not soundproof room in the hospital. Therefore, the measured lung sounds might have been contaminated by ambient noise. However, with regard to the good overall agreement and moderate interrater reliability for the presence of adventitious sounds, the results were almost equal for recordings from physicians and parents and were similar to what has been reported in other studies [9,17].

With this study, we have confirmed the feasibility of using the AIRDOC app to record lung sounds with quality, as most participants had at least 1 recording with quality per location when recordings were acquired by physicians (30/45, 67%) and when they were acquired by parents (27/45, 60%). The results of our study are consistent with those of previous studies in which lung sound recordings were classified by experts [9,17,19]. For instance, the fact that recordings taken at the trachea have shown greater proportions of agreement (99%) and greater proportions of quality lung sounds (99%) has also been documented [9] and might be attributable to sounds having higher frequencies at this location, as the trachea has fewer tissues, which results in less filtering of sound signals [20,21]. These characteristics suggest that the trachea may be one of the best locations for the parental monitoring of respiratory status in real life. However, future studies with larger samples need to clarify whether adventitious sounds heard in the trachea are of clinical relevance for timely shared decisions. In adult patients with chronic obstructive pulmonary disease, sounds from this location could be used to predict exacerbations 5 days in advance [22].

Although only 13.4% (34/253) and 11.7% (31/266) of the physicians’ and parents’ recordings, respectively, had an identifiable adventitious sound, the percentage of participants with at least 1 lung sound recording with adventitious sounds (19/45, 42%) was similar to those found in previous works (35% in the study by Ferreira-Cardoso et al [9] and 28% in a study by Aviles-Solis et al [23]). The presence of adventitious sounds in the children without chronic respiratory diseases could be attributed to respiratory infections, as the recordings were made during the season with the highest incidence of respiratory infections. In addition, some of these participants were being followed up in the outpatient department due to a suspicion of a respiratory disease that had not yet been established, even though the presence of adventitious sounds has also been documented in healthy people [24,25]. The small difference between the presence of adventitious sounds in physicians’ recordings and that in parents’ recordings could be explained by the fact that the auscultations occurred some minutes apart rather than simultaneously (ie, the respiratory cycles differed among recordings) [26], which is a limitation of our study and should be addressed in further studies.

Although the parents’ contact with the app was brief, they provided positive feedback on the auscultation feature with regard to its ease of use and their willingness to use it as a tool for communicating with the physician. Features related to an app’s interface (eg, reduced number of screens and limited manual data entry) and communication with the health care team are among the features that are most valued by patients [27]. Parents’ willingness to recommend the app to others was also high and similar to what has been reported in previous studies of asthma apps [28,29]. These findings are encouraging for the continued development of the AIRDOC app.

### Limitations

This study has some limitations that need to be acknowledged. The results obtained from parents’ recordings may not be generalizable to the real-life use of the app. Parents’ performance in recording lung sounds was influenced by the fact that they were able to watch a demonstration of the procedure by observing physicians beforehand, and during parents’ performance, physicians were able to give advice (how to press the smartphone, how to hold the smartphone, and no talking). However, the AIRDOC app is being developed for monitoring purposes in personalized follow-up care; therefore, demonstrations are being planned for parents. Another possible limitation was the use of the physicians’ smartphones by the parents. We were aware of the possible effects of using an unfamiliar device; however, the decision to avoid the time-consuming process of installing the app on parents’ smartphones was made, considering the internet connectivity limitations in the outpatient department. Additionally, comparing the smartphone auscultations performed by parents and physicians who used the same device allowed us to avoid the effects of differences among various embedded microphones. In the future, the feasibility of parents using the app should be evaluated in real life, with parents using their smartphones outside the clinical context. For this purpose, clear instructions on how to perform the auscultations should be made available in the app. With older children, we could have tested the self-recording of lung sounds, as they were old enough to manage their own diseases and treatment plans, but this would have increased the duration of the procedure, and it should be noted that data collection took place during routine medical visits. The self-recording of lung sounds by older children should however be carried out in further studies. Furthermore, the classification of adventitious sounds was based on broad classes (crackles and wheezes), without an attempt to distinguish subtypes, such as coarse crackles, fine crackles, high-pitch wheezes, and low-pitch wheezes. This decision was made in light of previous agreement studies, which showed that a broad classification was more reliable among experts than more detailed descriptions. We recognize that adventitious sound features are relevant to clinical decision-making, but it is sometimes very difficult for the human ear to discriminate these features [17]. The development of automated lung sound analysis methods will help us to overcome this limitation [30]. In addition, the proportions of recordings with crackles and recordings with wheezes were not analyzed separately; instead, our results are based on the proportions of recordings with adventitious lung sounds. This strategy was related to the small sample size and the small proportion of adventitious sounds (ie, crackles) in the recordings. The small sample size also hindered the comparison of smartphone auscultation performance among diagnosis groups; therefore, the project will continue to recruit children to strengthen the current findings. Another limitation is that other factors that may have been assessed by the physicians during the medical visits, such as respiratory rate, thoracic perimeter, and abdominal perimeter, were not included in our data collection. These parameters may be related to lung sound features and should be considered in the future.

### Conclusions

The main findings suggest that lung auscultation via a smartphone built-in microphone is feasible when performed by parents, as they can record lung sounds with quality and can successfully capture adventitious sounds. This study also shows that parents are willing to use this technology in real life to provide feedback to physicians. Thus, smartphone lung auscultation can potentially be performed by parents to monitor children’s respiratory status in real life. Additional research is needed to develop this technology further.

## Supplementary material

10.2196/52540 Multimedia Appendix 1 Participants’ (N=46) characteristics.

10.2196/52540 Multimedia Appendix 2 Comparisons of the agreement for and the proportions of physicians’ and parents’ quality recordings.

## Abbreviations

STROBE: Strengthening the Reporting of Observational Studies in Epidemiology
