# Supplementary material for: Feasibility and Acceptability of Pediatric Smartphone Lung Auscultation by Parents: Cross-Sectional Study
Source: JMIR Pediatr Parent. 2024 Apr 8;7:e52540. doi: 10.2196/52540 (PMC11024396; doi:10.2196/52540)
Supplement: Multimedia Appendix 1 [file pediatrics-v7-e52540-s001.docx]

**Multimedia Appendix 1.** Participants’ (N=46) characteristics.

| Characteristics | Total | Asthma | Other  Respiratory  Diseases | No  Respiratory  Diseases | p |
| --- | --- | --- | --- | --- | --- |
| Subjects, n (%) | 46 (100%) | 24 (52%) | 15 (33%) | 7 (15%) | <.001 |
| Male, n (%) | 33 (72%) | 20 (83%) | 8 (53%) | 5 (1%) | <.001 |
| Age, mean [SD] y | 11.3 [3.1] | 11.0 [3.2] | 12.0 [3.0] | 10.3 [3.1] | .135 |
| Height, mean [SD] cm | 148.2 [16.6] | 147.0 [16.7] | 151.8 [18.2] | 143.9 [11.5] | .197 |
| Weight, median [Q1-Q3] Kg | 44.2 [30.8-56.2] | 41.8 [29.1- 56.8] | 49.0 [32.0-58.0] | 40.8 [29.8-52.7] | .009 |
| BMI, median [Q1-Q3] Kg/m^2^ | 19.4 [16.4-21.5] | 19.6 [16.4-21.6] | 19.0 [15.4-21.4] | 18.9 [16.9-23.1] | <.001 |

Q1-Q3: interquartile range between the 25th and the 75th percentile. SD, standard deviation. BMI, body mass index.
