# Supplementary material for: Feasibility and Acceptability of Pediatric Smartphone Lung Auscultation by Parents: Cross-Sectional Study
Source: JMIR Pediatr Parent. 2024 Apr 8;7:e52540. doi: 10.2196/52540 (PMC11024396; doi:10.2196/52540)
Supplement: Multimedia Appendix 2 [file pediatrics-v7-e52540-s002.docx]

**Multimedia Appendix 2.** Comparisons of the agreement for and the proportions of physicians’ and parents’ quality recordings.

|  |  | **Comparison** | | | **Chi-Square** |
| --- | --- | --- | --- | --- | --- |
| Physician | Agreement | Auscultation location overall comparison | | | <0.001* |
|  |  | Trachea | Right Anterior Chest | | <0.001* |
|  |  | Trachea | Right Posterior Base | | <0.001* |
|  |  | Trachea | Left Posterior Base | | 0.042 |
|  |  | Right Anterior Chest | Right Posterior Base | | 0.335 |
|  |  | Right Anterior Chest | Left Posterior Base | | 0.013 |
|  |  | Right Posterior Base | Left Posterior Base | | 0.104 |
|  |  |  |  |  |  |
|  | Proportion of quality | Auscultation location overall comparison | | | 0.015* |
|  |  | Trachea | Right Anterior Chest | | <0.001* |
|  |  | Trachea | Right Posterior Base | | 0.004* |
|  |  | Trachea | Left Posterior Base | | <0.001* |
|  |  | Right Anterior Chest | Right Posterior Base | | 0.075 |
|  |  | Right Anterior Chest | Left Posterior Base | | 0.443 |
|  |  | Right Posterior Base | Left Posterior Base | | 0.315 |
| Parent | Agreement | Auscultation location overall comparison | | | 0.109 |
|  | Proportion of quality |  |  | |  |
|  |  | Auscultation location overall comparison | | | 0.006* |
|  |  | Trachea | | Right Anterior Chest | 0.019 |
|  |  | Trachea | | Right Posterior Base | 0.001* |
|  |  | Trachea | | Left Posterior Base | 0.326 |
|  |  | Right Anterior Chest | | Right Posterior Base | 0.355 |
|  |  | Right Anterior Chest | | Left Posterior Base | 0.162 |
|  |  | Right Posterior Base | | Left Posterior Base | 0.022 |

* A statistically significant difference was found.
